# Distribution and Diversity of *Beauveria* in Boreal Forests of Northern European Russia

**DOI:** 10.3390/microorganisms9071409

**Published:** 2021-06-29

**Authors:** Igor A. Kazartsev, Georgy R. Lednev

**Affiliations:** Laboratory of Mycology and Phytopathology, Laboratory of Microbiological Plant Protection, All-Russian Institute of Plant Protection (FSBSI VIZR), Pushkin, 196608 St. Petersburg, Russia; georgijled@mail.ru

**Keywords:** *Beauveria*, boreal forest, Cordycipitaceae, ecology, phylogenetic analysis, entomopathogenic fungi, haplotypic diversity

## Abstract

The distribution and genetic diversity of 91 of *Beauveria* isolates collected during a long-term survey in boreal forests of northern European Russia was studied. Based on morphological and sequence analysis of TEF and Bloc loci, three *Beauveria* spp. were identified: *B. pseudobassiana*, *B. bassiana,* and *B. caledonica*, with abundance of 81, 11, and 8%, respectively. Through multilocus sequencing, four haplotypes of *B. bassiana* and two haplotypes of *B. caledonica* were detected. Twelve haplotypes of *B. pseudobassiana* with non-random distribution were identified. Two haplotypes of *B. pseudobassiana* were the most abundant and widespread occurring across the whole study area, whereas others tended to be more specific to either the north or south of the study area, indicating the presence of different subpopulations. For further analysis of these putative subpopulations, southern and northern areas were separated along the boundary of the Köppen–Geiger climate zones (dfb and dfc), and the genetic structure was examined by analysis of molecular variance and spatial autocorrelation. Molecular evidence of intraspecific recombination of *B. pseudobassiana* and *B. bassiana* across northern European Russia area was indicated.

## 1. Introduction

The genus *Beauveria* includes globally ubiquitous soilborne arthropod-pathogenic pleomorphic fungi belonging to Cordycipitaceae (Hypocreales) with high ecological and economical importance [1,2,3]. These fungi are important in the regulation of insect populations in natural environments, also occurring as saprotrophs and plant endophytes [4]. *Beauveria bassiana* (Bals.-Criv.) Vuill. and *Beauveria brongniartii* (Sacc.) Petch are the most common fungi in mycoinsecticide formulations used to suppress various agricultural and forest insect pests [5]. *Beauveria* spp. produce many secondary metabolites, which offer a promise for biotechnology [6,7].

The history of research on this fungal group started in the early nineteenth century with the work of Agostino Bassi [8]. He was the first to show the fungal etiology of the insect white muscardine disease. In honor of Bassi, this fungus received the name *Botrytis bassiana* Bals.-Criv. Vuillemin established the new genus *Beauveria* and made *B. bassiana* the type species. During the twentieth century, it became clear that many species, which were previously assigned to *Clavaria*, *Botrytis*, *Sporotrichum*, *Isaria*, *Tritirachium*, and others, should be transferred to *Beauveria* [9]. On the opposite, other species were excluded from this genus. De Hoog [10] then reduced the genus to only three species, *В. bassiana*, *B. brongniartii*, and *Beauveria alba* (Limber) Saccas (currently *Parengyodontium album* (Limber) C.C. Tsang et al.). Subsequently, Rehner et al. [1] divided *B. bassiana* s.l. and *B. brongniartii* s.l. into several cryptic species based on the three nuclear genes (TEF, RPB1, and RPB2) and the nuclear intergenic region (Bloc) phylogeny. Consequently, the understanding of the taxonomic structure of *Beauveria* has been significantly broadened. Many species have since been described with the aid of molecular methods [11,12,13,14,15,16,17]. The most recent phylogenetic studies led to *Beauveria*, previously only composed of species known from asexual morphs, being expanded to include taxa previously described by sexual morphs as *Cordyceps* [16,18].

The majority of studies concerning the community diversity, ecology, and population structure of *Beauveria* were focused on *B. bassiana* s.l. These studies have revealed high genetic diversity within local geographical populations using a range of molecular methods, including RFLP, AFLP, DGGE, and microsatellite markers [19,20,21,22,23]. However, neither these methods nor ITS sequencing has provided sufficiently comparable data to infer interspecies and population genetic structures within *Beauveria*. Recent studies have been more consistent with the sequencing of nuclear intergenic locus Bloc being effectively mandatory for the determination of *Beauveria* phylogeny [3,24,25]. Most of *Beauveria* spp. have been reported from particular geographical regions, but a few species (e.g., *B. bassiana* and *Beauveria pseudobassiana* S.A. Rehner and Humber) have worldwide distributions. Several studies have demonstrated a strong association of *B. bassiana* with agricultural and *B. pseudobassiana* with forest ecosystems [24,25].

The natural insect host range of *Beauveria* spp. includes 17 orders of Arthropoda [3,26,27]. A number of studies have shown the lack of significant relationships between genotype and host specificity in *Beauveria* [28,29,30,31,32]. However, recent studies with comparative whole-genome sequencing have indicated that several genes may underpin virulence towards the hosts during the infection process [33,34].

In comparison to the long history of global research on inter- and intraspecific diversity in *Beauveria*, there have been considerably fewer studies in Russia (and the former USSR). Evlakhova [35] reported the widespread occurrence of *B. bassiana* s.l. from the Baltic regions to Sakhalin and from the Kola Peninsula to Transcaucasia. Many previous studies were conducted using obsolete species names and, before the recognition of cryptic species in *B. bassiana* s.l. and *B. brongniartii* s.l., based on molecular markers. Therefore, there is a need to reevaluate the presence of species and intraspecific forms of *Beauveria* according to modern taxonomic understanding using contemporary research methods. Although there has been limited recent work on *Beauveria* phylogeny, more progress has been made on screening virulent strains for biocontrol purposes and their adoption as bioinsecticides [35,36,37].

In Russia, systematic investigations of genetic diversity in *Beauveria* were undertaken independently in some but not all geographical regions; therefore, the available information is clearly incomplete. Consequently, the goal of this study was to contribute to the information on the species’ and interspecies’ genetic diversity within *Beauveria* in northern European Russia (NER) because no previous study with material from this region has been conducted with specific molecular markers. We hypothesized that *B. pseudobassiana* and *B. bassiana* have a non-random geographical distribution and genetic structure across this focus region.

## 2. Materials and Methods

### 2.1. Specimen Collection and Fungal Isolation

This study combined data from three field surveys conducted in August 2017, 2018, and 2019 in boreal forest ecosystems of NER. During these surveys, insect cadavers with signs of mycosis were collected from under the bark of living trees, logs and stumps, and from litter around trees. Ninety-one fungal isolates were collected from infected host insects from 31 locations. In 2017, collections were made in the Republic of Karelia and the Leningrad region. The target areas shifted to the Vologda, Pskov, and Novgorod regions in 2018 and the Republic of Karelia and Arkhangelsk and Vologda regions in 2019. In addition, two accessions from the Pskov region, originally collected in 2013, were included in the analysis. The distance between the most distant locations was over 1000 km. A detailed list of the collections and Genbank accession numbers of sequences are given in Table S1. The identification of accompanying entomopathogenic species from other genera was also undertaken as detailed in Table S2.

Fungal isolation was done by transferring conidia and mycelium fragments from insect cadavers on Sabouraud dextrose agar (SDA, Difco, Detroit, MI, USA) with a sterile inoculating needle. When no conidia were observed, the stimulation of fungal development was done by the moist-chamber technique. Initial identification of *Beauveria* spp. and entomopathogens was performed based on the examination of macro- and micromorphological features.

### 2.2. DNA Extraction, PCR Amplification, and Sequencing

Genomic DNA was prepared from homogenized mycelium in 1.5 mL Eppendorf tubes using CTAB/chloroform DNA extraction procedure [38]. DNA probes were resuspended in TE buffer and stored at −20 °C. Primers for partial amplification of translation elongation factor-1a (TEF) were 983F and 1567R [32]. Primers B5.1F and B3.1R were used to amplify the nuclear intergenic region, Bloc [39].

All PCR reactions were performed in a final volume of 25 μL containing 2.5 μL 10× PCR buffer, 0.5 μL dNTP mix (10 mM), 0.5 μL of each primer (10 μM), 0.15 μL of Taq DNA polymerase (5 U/μL, Qiagen, Hilden, Germany), 1 μL of genomic DNA, and 19.85 μL diH_2_O. Parameters of DNA amplification were as in Rehner and Buckley [32] and Rehner et al. [39].

The PCR product quality was assessed by electrophoresis on an agarose gel (1%) stained with ethidium bromide in 1× TBE buffer. The bands chosen for sequencing were excised and then purified with silica particles [40]. The Sanger sequencing of PCR products was performed in both directions using a capillary DNA sequencer ABI 3500 (Applied Biosystems, Foster City, CA, USA).

### 2.3. Bioinformatic Analyses

Nucleotide sequences obtained were assembled and manually edited using Vector NTI Advance 11.5.1 software (Life Technologies, Carlsbad, CA, USA). Sequences were aligned using algorithm MUSCLE [41] in bioinformatics software tool MEGA X [42]. All sequences were trimmed evenly to eliminate the variation in sequence length. After alignment, the concatenation of TEF and Bloc sequences was conducted in SequenceMatrix 1.7.3 for further multilocus analysis [43]. Appropriate nucleotide substitution models were identified with jModelTest v. 2.1.10 basing on the corrected Akaike information criterion (AICc) [44,45]. Models K2, T92 + G, and HKY + F + G4 were chosen for TEF, Bloc, and concatenated sequences, respectively [46,47,48]. Then, a maximum likelihood analysis with 1000 bootstrap replicates was performed on the TEF and Bloc datasets to obtain phylogenetic trees using MEGA X. Function cophylo from R package “phytools” was used to generate a co-phylogenetic plot of TEF and Bloc trees (in R v. 4.0.3) [49,50]. Phylogenetic reconstruction with concatenated sequences was conducted in IQ-tree 2.1.2 with 1000 ultrafast bootstrap approximation [51,52].

The number of haplotypes and haplotype diversity (Hd) were calculated using DnaSP v. 5.10 [53,54]. Species richness was estimated as the number of different *Beauveria* spp. The relative species abundance was considered as the percentage of isolates from each fungal species to the total number of fungal isolates. The species’ relative occurrence was estimated as the percentage of the locations in which a certain species was detected to the total number of locations.

*Beauveria* spp. isolates had a varying heterogeneous genetic structure in the southern and northern parts of the study area. Therefore, the boundary between the two Köppen-Geiger climate zones [55] was used to investigate this structure. Appropriate coordinates were downloaded from the World Maps of Köppen-Geiger Climate Classification (http://koeppen-geiger.vu-wien.ac.at/present.htm (accessed on 4 March 2021)). The south area was in the Köppen-Geiger zone dfb with a warm-summer-humid continental climate, and the north area was in zone dfc with a subarctic climate. Arlequin 3.5 was used to test the hypothesis that variability in the genetic structure of *B. pseudobassiana* was related to climate zones (dfb and dfc) based on the hierarchical analysis of molecular variance (AMOVA) [56].

SplitsTree 4.10 was used to find evidence of recombination based on phylogenetic incompatibilities of polymorphic sites along with concatenated sequences with PHI test [57,58]. The test was performed with the default settings of a window size of 100 and k = 3. In addition, the phylogenetic diversity (the sum of the weights for all splits) was estimated with SplitsTree 4.10 [59].

The standardized index of association (IAs) indicating the degree of linkage disequilibrium among different loci and significance of the null hypothesis was calculated under Monte Carlo simulation (10,000 iterations) with LIAN 3.7 [60]. Indices were computed for each fungal species on complete and clone-corrected datasets. Clone-corrected datasets were assembled by removing replicates of the same haplotype since repetition due to clonality can lead to the detection of linkage disequilibrium and consequently can affect the ability to detect recombination among isolates [61].

We used the global multilocus spatial autocorrelation analysis implemented in GenAlEx 6.5 [62] to describe the genetic structure across an entire studied region [63]. The autocorrelation coefficient (r) provides a measure of the genetic similarity between pairs of individuals whose geographic separation falls within the specified distance class. Pairwise individual-by-individual genetic and geographic distances were used to calculate r for predefined 18 distance classes (class size 50 km). Spatial genetic autocorrelograms were created by plotting r values as a function of distance. Tests for statistical significance were performed using 10,000 random permutations of the individuals and 10,000 bootstraps for estimates of r. Correlation values outside the confidence interval are considered to be statistically significant at *p* < 0.05.

The geographical mapping of the sampling locations and species/haplotypes distribution were done with the R packages “maps” [64], “ggplot2” [65], and “plotrix” [66]. Rarefaction curves were generated with the R package “iNEXT” [67,68].

## 3. Results

### 3.1. Species Abundance, Occurrence, and Host Specificity

**Figure 1:**
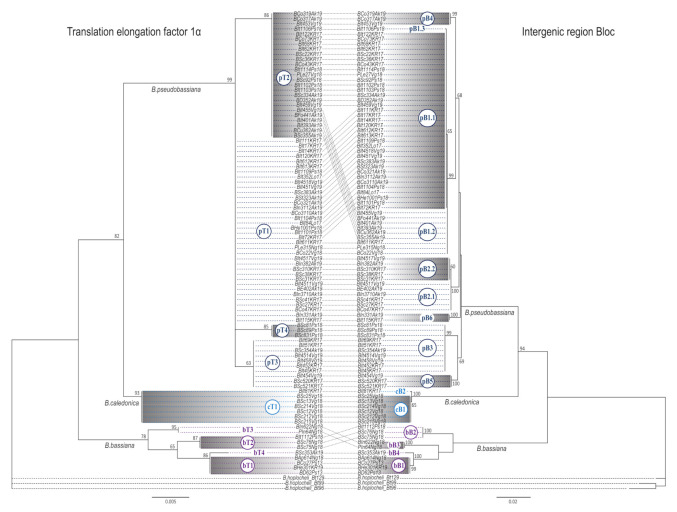
Phylogenetic trees of *Beauveria* spp. based on the maximum likelihood (ML) analyses of two locus (TEF gene and nuclear intergenic locus Bloc). ML bootstrap values (>50%) are shown at the nodes. Gray and white areas separate alternating alleles. Alleles of each locus are designated with individual index numbers. *Beauveria* species epithets *pseudobassiana*, *bassiana*, and *caledonica* are indicated with lowercase letters (p, b, and c), TEF and Bloc alleles with capital letters (T and B), and then numbers in order of abundance. *Beauveria pseudobassiana* was divided into six clades by Bloc, with the subsequent hierarchical allelic assignment.

**Figure 2:**
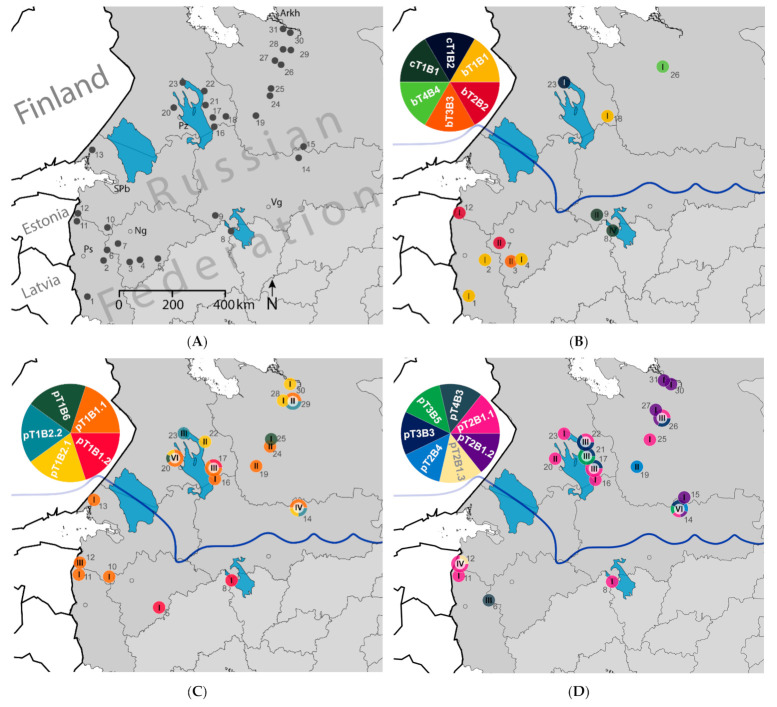
Distribution of *Beauveria* spp. And haplotypes across northern European Russia. (**A**) Geographical map and specimens’ locations numbered with respect to Table S1. Abbreviations: Arkh—Arkhangelsk, Vg—Vologda, Ng—Novgorod, Ps—Pskov, SPb—St. Petersburg, Pz—Petrozavodsk. (**B**) Distribution of *B. bassiana* (bT1B1 to bT4B4) and *B. caledonica* (cT1B1 to cT1B2) haplotypes. (**C**) Distribution of *B. pseudobassiana* haplotypes (pT1B1.1 topT1B6). (**D**) Distribution of *B. pseudobassiana* haplotypes (pT2B1.1 to pT4B3). The haplotypes’ designations are derived from Figure 1. Roman numerals correspond to isolating the quantity at that location. The blue line indicates the boundary between the southern and northern climate zones (dfb and dfc) according to the Köppen-Geiger climate classification.

Overall, specimens (insect host cadavers) from 31 locations of northern European Russia were collected, and 91 fungal isolates were purified and sequenced (Figure 1 and Figure 2A, Table S1). Species richness of *Beauveria* was limited to three species: *B. pseudobassiana*, *B. bassiana,* and *Beauveria caledonica* Bissett and Widden. *Beauveria pseudobassiana* was the most frequently collected and abundant species (occurrence 77% and abundance 81%). *Beauveria bassiana* and *B. caledonica* were less dominant, with abundance of 11 and 8%, respectively.

*Beauveria bassiana* was collected in eight locations (occurrence 26%), usually separate from other species. However, in two locations (locations 12 and 26), *B. bassiana* was obtained from Coleoptera (Scolytinae) concurrently with *B. pseudobassiana* (Figure 2, Table S1). *Beauveria caledonica* during the present surveys was detected in only three locations (occurrence 10%). Therefore, it could not be included in all statistical analyses.

The uneven distribution of *Beauveria* spp. across the study area was recorded (Figure 2B–D). Most *B. bassiana* isolates (80%) were collected from the southern part of the study area (Köppen-Geiger zone dfb). In contrast, most *B. pseudobassiana* isolates (77%) were collected in the north (Köppen-Geiger zone dfc). Six *B. caledonica* isolates were collected from locations 9 and 8 in zone dfb, and only one (location 23) was from zone dfc.

About 88% of *B. pseudobassiana* isolates were collected from Coleoptera; among them, a significant portion was isolated from hosts in the subfamily Scolytinae (73%). Less frequently, *B. pseudobassiana* was isolated from Lepidoptera (3%), Hemiptera (1%), Hymenoptera (1%), Diptera (1%), and undefined Insecta (5%). *Beauveria bassiana* was isolated from Coleoptera (50%), Hemiptera (10%), Hymenoptera (10%), Diptera (10%), and undefined Insecta (30%). *Beauveria caledonica* was only isolated from bark beetles (Scolytinae).

In addition to *Beauveria* spp., entomopathogenic fungi from other taxonomic groups were identified in the same locations: *Akanthomyces* cf. *attenuatus* (Zare and W. Gams) Spatafora, Kepler, and B. Shrestha (locations 16, 19, 20, 21, and 25), *Cordyceps bifusispora* O.E. Erikss (location 5), *Cordyceps militaris* (L.) Fr. (locations 5, 19, 24, and 27), *Cordyceps farinosa* (Holmsk.) Kepler, B. Shrestha, and Spatafora (locations 10, 19, 20, 26, and 27), *Metarhizium* sp. (location 8), *Ophiocordyceps entomorrhiza* (Dicks.) G.H. Sung, J.M. Sung, Hywel-Jones, and Spatafora (location 29) and *Tolypocladium* sp. (location 14; Table S2).

**Figure 3:**
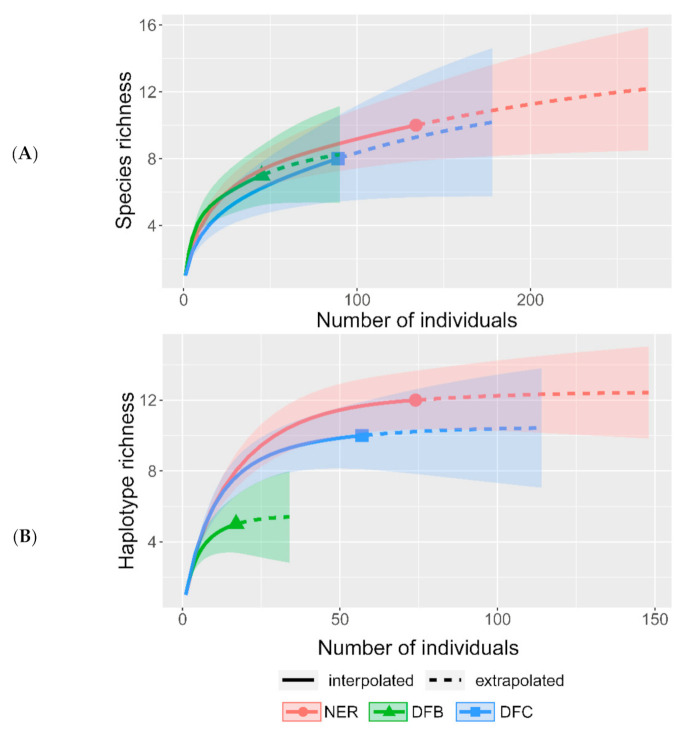
Rarefaction curves for total species richness (*Beauveria* spp. and other arthropod-pathogenic species isolated in this study; (**A**) and for *B. pseudobassiana* haplotype richness (**B**) observed in northern European Russia (NER) and the southern (dfb) and northern (dfc) climate zones. Rarefaction curves for *B. bassiana* and *B. caledonica* could not be obtained due to insufficient data. Shaded areas on plots correspond to 95% confidence intervals.

The uneven intensity of sampling for *Beauveria* spp. across NER could lead to its underestimation in either zone dfb or dfc. To determine the completeness of the available datasets, several individual rarefaction curves were generated for (1) all identified species including entomopathogenic fungi from other genera (Figure 3A, Tables S1 and S2), (2) the three *Beauveria* spp. (Figure S1 and Table S1), and (3) all *B. pseudobassiana* haplotypes (Figure 3B and Table S1). Rarefaction curves for the dataset with entomopathogenic fungi from other genera indicated a slight underestimation but were beyond the linear ranges. Rarefaction curves for *Beauveria* spp. and *B. pseudobassiana* haplotypes reached the asymptote and indicated the adequacy of sampling intensity across NER, as well as zones dfb and dfc. This indicates that a high proportion of *Beauveria* spp. and *B. pseudobassiana* haplotypes were collected in the present survey.

### 3.2. Genetic Diversity

The intraspecific diversity of *B. pseudobassiana* was sufficiently greater than for the other two species. Total Hd for *B. pseudobassiana* isolates across all locations reached 0.638 (TEF), 0.738 (Bloc), and 0.863 (multilocus sequences; MLS; Table S3). Total Hd for *B. bassiana* isolates was 0.778 for each locus and MLS data. For *B. caledonica*, total Hd was 0.286 for Bloc and MLS and 0 for TEF since the latter was represented by only one allele. Total phylogenetic diversity based on MLS data was estimated as 0.033 for *B. pseudobassiana*, 0.054 for *B. bassiana*, and 0.001 for *B. caledonica*. The MLS phylogenetic tree of *Beauveria* spp. is presented in Figure S2.

*Beauveria bassiana* was represented by four TEF alleles (bT1, bT2, bT3, and bT4) and four Bloc alleles (bB1, bB2, bB3, and bB4; Figure 1). The most abundant haplotype was bT1B1 representing 40% of total *B. bassiana* isolates. *Beauveria caledonica* was represented by one TEF allele (cT1) and two Bloc alleles (cB1 and cB2). Each of the two *B. caledonica* haplotypes (cT1B1 and cT1B2) were found in separate climate zones.

The most representative data were obtained for *B. pseudobassiana* isolates, and the genetic structure of this species was obtained in more detail. *Beauveria pseudobassiana* had four distinguishable alleles by TEF, which were marked as pT1, pT2, pT3, and pT4, according to their abundance (Figure 1). Alleles pT1 and pT2 were predominant and were represented by 49 and 34% of isolates, respectively. They were widely distributed over the study area. Alleles pT3 and pT4 were significantly less abundant. Allele pT3 (14%) was detected only in the north of the study area, while pT4 (4%) was found only in the south (a single location of the Pskov region).

The intergenic region Bloc of *B. pseudobassiana* allowed the identification of six clades, which were designated as pB1–pB6. Within all clades, nine individual alleles were observed and numbered with respect to their clade (e.g., alleles pB1.1, pB1.2, and pB1.3 corresponded to clade pB1). The most predominant allele was pB1.1, occurring in 47% of isolates. Less frequent were pB1.2 and pB3, with an occurrence within isolates of 12 and 14%, respectively. All other six Bloc alleles had an occurrence of less than 8% each and were detected in up to six isolates. Alleles pB1.1, pB1.2, and pB3 were more or less evenly distributed, whereas alleles pB2.1, pB2.2, pB4, pB5, and pB6 were found only in isolates from the northern zone. Allele pB1.3 was found in a single isolate from the Pskov region.

Haplotypes with a combination of pT1B1.1 and pT2B1.1 were the most frequent (27 and 20%, respectively) and widespread. Haplotype pT1B1.2 had a uniform distribution and was detected in the Republic of Karelia and the Novgorod and Vologda regions but was quite rare. Since alleles pB2.1, pB2.2, pB4, pB5, and pB6 were found only in the northern zone, all occurred combinations with TEF alleles (pT1, pT2, and pT3) were detected also strictly in the northern zone.

It is worth noting that several haplotypes were collected from one location, revealing the highly variable heterogeneous haplotype structure of *B. pseudobassiana*. For example, eight haplotypes were found in location 14 and four haplotypes in locations 17 and 20, respectively.

Table 1 presents the result of AMOVA used to evaluate the population genetic structure of *B. pseudobassiana* in two climate zones (dfb and dfc), with each population corresponding to one location (Table 1). This partitioning scheme showed a low genetic differentiation between the dfb and dfc zones (2.5%), with the most variation observed within populations (79.2%). The variation derived from populations within each climate zone was moderate (18.3%) and reflected the heterogeneous haplotype structure at a single location, as previously mentioned. The fixation index between populations within groups was significant (FSC = 0.19; *p* = 0.007), while it was lower and not significant between groups (FCT = 0.03; *p* = 0.215).

To avoid the impact of heterogeneity within locations, we increased the size of the populations and linked them to the administrative division (Vologda region locations were allocated to the northern and southern zones according to the climate boundary). In this case, the fixation index between the groups was significant (FCT = 0.07; *p* = 0.029) and exceeded the fixation index within populations (FSC = −0.05; *p* = 0.781) showing a strong population genetic structure at the group scale. As expected, the grouping of populations only by administrative boundaries did not provide evidence of a genetic structure at this level (FCT = −0.10; *p* = 0.933).

**Figure 4:**
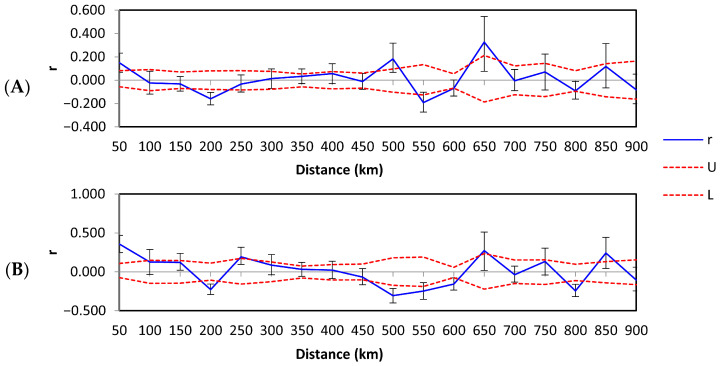
Spatial autocorrelograms of the genetic correlation coefficient (r) as a function of distance for *Beauveria pseudobassiana* across northern European Russia (**A**) for all haplotypes and (**B**) for all haplotypes with the exception of T1B1.1 and T2B1.1 (the most abundant) haplotypes in the dfc climate zone. Dashed lines represent the upper (U) and lower (L) bounds of the 95% confidence intervals about the null hypothesis of random distribution of haplotypes; error bars are bootstrapped 95% confidence intervals within each distance class.

Spatial autocorrelation for all *B. pseudobassiana* haplotypes across 18 distance classes showed a pattern of decreasing genetic relatedness up to 50 km, with an x-intercept of 93 km (Figure 4A, Table S4). In addition, positive and significant r values were observed at 500 and 650 km, reflecting the presence of non-random genetic structure at these distance classes. To understand the contribution of widespread haplotypes T1B1.1 and T2B1.1 to the spatial autocorrelation values, these haplotypes were separated from the dfc zone dataset to obtain another correlogram (Figure 4B). This resulted in the improvement of r values, which were still significant and positive up to 50 km, but the x-intercept was at 167 km. Further, positive r values still occurred at larger distance classes. The relationship between r and the distance was not significantly negative until 200 km on both plots. The correlograms showed the oscillation of high and low autocorrelation, indicating irregularity of specimen survey due to alternating areas of a high and low density of findings. Although, several signs of positive autocorrelation at a significant distance from each other might be evidence of several independent genetic aggregations.

### 3.3. Recombination

PHI test based on MLS data of 74 *B. pseudobassiana* isolates showed strong evidence of recombination (*p* = 0.007) between the haplotypes across the area of NER studied (Table 2, Table S3). Separate testing of 57 isolates from zone dfc resulted in significance (*p* = 0.010) and still reflected the recombination process (Table 2). However, the testing of the *B. pseudobassiana* population from zone dfb did not confirm that the recombination between individuals. Most likely, the results in zone dfb were due to insufficient data. Testing of 10 *B. bassiana* isolates provided evidence of recombination (*p* = 0.025). Further testing of *B. bassiana* in zone dfb did not confirm recombination. Testing of *B. bassiana* (dfc) and *B. caledonica* (all areas) did not provide evidence of recombination due to the insufficient informative characters (Table 2, Table S3).

The IAs, showing the degree of linkage between different loci, were calculated for each species separately on the complete and clone-corrected MLS datasets for all populations, as well as for populations in the two climate zones (Table 2, Table S3). IAs estimated for all *B. pseudobassiana* populations based on the complete dataset showed a moderate linkage disequilibrium significantly different from zero. Nevertheless, after the implementation of datasets with clone-corrected data, the null hypothesis was not rejected, indicating the linkage equilibrium and pointing to the potential existence of genetic recombination within populations of *B. pseudobassiana* (Table 2).

For the *B. bassiana* non-clone-corrected datasets, IAs differed significantly from zero in NER and zone dfb populations. This indicates the linkage between loci and thus the absence of sexual reproduction in populations. Other datasets for clone-corrected or zone dfc populations of *B. bassiana* were insufficient to be analyzed. Genetic recombination between *B. caledonica* isolates was not tested for the same reasons.

## 4. Discussion

The 91 fungal *Beauveria* isolates from northern European Russia included three *Beauveria* spp.: *B. bassiana*, *B. pseudobassiana,* and *B. caledonica*. Notably, *B. brongniartii* was not found; however, this species regularly appeared as *Beauveria tenella* (Sacc.) Siemaszko in the old Russian-language literature devoted to entomopathogens of boreal forests [35]. The existence of *B. caledonica* and the absence of *B. brongniartii* in the presented dataset, considering their similar morphological characteristics [1], may reflect incorrect species identification before molecular phylogeny was commonly used in mycological research. We suspect that most of *B. brongniartii* (=*B. tenella*) determinations in the earlier Russian literature may actually refer to *B. caledonica*, which was recently detected in Russia and considered to be quite rare [69].

Intraspecific heterogeneity of *Beauveria* spp. in the study area made it necessary to more closely examine the data to seek an explanation for this phenomenon. Analysis of biodiversity according to artificial administrative boundaries has widely been used but was not suitable in terms of ecology, especially if we can define any gradient of environmental factor. For these purposes, we divided the survey area north–south by climate zone (dfb and dfc) according to the Köppen-Geiger climate classification. The division was between the warm-summer-humid continental climate and subarctic climate. However, it is possible that a division of the southern and middle boreal forests would be more informative, even though this boundary is relatively close to the boundary between dfb and dfc.

Based on the climate zone division, an uneven distribution of *B. bassiana* and *B. pseudobassiana* was revealed. Earlier studies indicated a strong association of *B. bassiana* with agricultural and *B. pseudobassiana* with forest ecosystems [24,25]. Our findings were consistent with these reports as the major species in the forest ecosystems were *B. pseudobassiana*, and the proportion of *B. bassiana* increased towards the southern (dfb) more agricultural areas (Figure 2B and Figure S3). Other studies have also indicated that *B. bassiana* is associated with the steppe zone, whereas *B. pseudobassiana* is more common in forest ecosystems [70]. Although only 10 *B. bassiana* isolates were obtained during the survey, they were collected from similar spectra of insect orders to *B. pseudobassiana*.

Sequencing of TEF and Bloc indicated significantly more haplotype richness in *B. pseudobassiana* (12 haplotypes per 74 isolates) than in *B. bassiana* (4 haplotypes per 10 isolates). In contrast, phylogenetic studies of *Beauveria* spp. isolated from soils in Slovakia based on sequencing analysis of ITS and Bloc loci found a limited genetic structure in *B. pseudobassiana* since one haplotype was found in 47 isolates. In contrast, only a slightly larger number of isolates (56) of *B. bassiana* had 15 haplotypes [24]. While studying the distribution and genetic diversity of *Beauveria* spp. in natural and agricultural ecosystems in China based on 641 isolates, 14 haplotypes of *B. pseudobassiana* and 13 haplotypes of *B. bassiana* were identified [25]. We found that several haplotypes detected in NER were more abundant and had wide distribution (T1B1.1 and T2B1.1), while others were less frequent and tended to occur mostly in the northern climate zone.

High haplotype diversity of *B. bassiana* in relatively small areas has been reported frequently [19,20,21,22,23]. For example, Wang et al. [19] characterized 13 haplotypes with the PCR-RFLP of the pr1 virulence-associated gene and 31 haplotypes with microsatellite markers among 77 *B. bassiana* isolates collected in pine forests in southeast China. Seven SSR markers used to study 81 *B. bassiana* isolates from *Lygus hesperus* of San Joaquin Valley (CA, USA) found three to nine distinct alleles [21]. A comparison of such studies is complicated because they were conducted with different molecular approaches and before the division of *B. bassiana* s.l. into several phylogenetic species.

A teleomorph–anamorph connection was previously reported for *B. brongniartii* [71]. Currently, there are ten members of genus *Beauveria*, including *B. pseudobassiana,* that also have a confirmed sexual reproduction cycle [3,14,16,18,72]. Consistently, our testing of *B. bassiana* and *B. pseudobassiana* isolates revealed that sexual or parasexual recombination across NER is possible. Based on our experience and multi-year surveys, there was no evidence of a teleomorph of these species that could be confirmed from the specimens collected. *Cordyceps* teleomorphs within the *Beauveria* clade have been mainly reported from East Asia or Amazonia [18]. Whether the putative recombination detected is sexual or parasexual, e.g., during infection of a single arthropod host by several fungal haplotypes, remains to be resolved.

## 5. Conclusions

The boreal forests of northern European Russia are characterized by low *Beauveria* species diversity, with only three species (*B. caledonica*, *B. bassiana,* and *B. pseudobassiana*) confirmed by molecular methods. Despite earlier reports of *B. brongniartii* in northern European Russia, it was not recollected in this more comprehensive study, so these are considered doubtful. Consistent with earlier findings, *B. pseudobassiana* was more abundant in forest ecosystems, and *B. bassiana* increased in occurrence towards the more agricultural areas sampled. Significant haplotype diversity in *B. pseudobassiana* revealed widespread haplotypes and haplotypes that tended to occur in the more northerly areas. Despite the absence of teleomorph specimens in this study, there was molecular clear evidence of intraspecific recombination in *B. pseudobassiana* and, to a lesser extent, in *B. bassiana*.

## Figures and Tables

**Table 1:** Analysis of molecular variance for *B. pseudobassiana* using MLS data for different grouping variants. Data were partitioned to test the effect of the genetic structure in the Köppen-Geiger climate zones (dfb and dfc) of northern European Russia by population sizes and administrative boundaries.

Grouping	Source of Variation	DF ^1^	SS	V (%)
**Group 1:** 7 populations from locations 5, 6, 8, and 10–13 **Group 2:** 17 populations from locations 14–17 and 19–31	Between groups	1	14.86	2.5
	Between populations within groups	22	193.80	18.3
	Within populations	50	262.32	79.2
	Total	73	470.99	-
***F**_SC_*: 0.19 (*p* = 0.007), ***F**_ST_*: 0.21 (*p* = 0.004), and ***F**_CT_*: 0.03 (*p* = 0.215)				
**Group 1:** Leningrad Reg. (locations 10 and 13); Pskov Reg. (locations 6, 11, and 12); Novgorod Reg. (location 5); Vologda Reg. (south, location 8) **Group 2:** Arkhangelsk Reg. (locations 15, 19, and 24–31); R. of Karelia (locations 16, 17, and 20–23); Vologda Reg. (north, location 14)	Between groups	1	14.86	7.0
	Between populations within groups	5	18.19	−5.0
	Within populations	67	437.94	98.0
	Total	73	470.99	-
***F**_SC_*: −0.05 (*p* = 0.781), ***F**_ST_*: 0.02 (*p* = 0.648), and ***F**_CT_*: 0.07 (*p* = 0.029)				
**Group 1:** Arkhangelsk Reg. (locations 15, 19, and 24–31) **Group 2:** R. of Karelia (locations 16, 17, and 20–23) **Group 3:** Vologda Reg. (locations 8 and 14) **Group 4:** Pskov Reg. (locations 6, 11, and 12) **Group 5:** Novgorod Reg.(location 5) **Group 6:** Leningrad Reg. (locations 10 and 13)	Between groups	5	25.68	−9.6
	Between populations within groups	18	182.98	27.5
	Within populations	50	262.32	82.1
	Total	73	470.99	-
***F**_SC_*: 0.25 (*p* = 0.001), ***F**_ST_*: 0.18 (*p* = 0.005), and ***F**_CT_*: −0.10 (*p* = 0.933)				

^1^ DF, degrees of freedom; SS, sum of squares; V (%), percent variation; R, Republic; Reg, Region.

**Table 2:** Estimates of recombination of *Beauveria bassiana* and *B. pseudobassiana*. Estimates for *B. caledonica* were not possible due to insufficient informative characters.

Parameter	Dfb ^1^	Dfc	NER
*Beauveria pseudobassiana*			
PHI test ^2^	no recombination	recombination **	recombination **
IAs NCC ^3^	0.2928 **	0.2290 ***	0.1970 ***
IAs CC	−0.0667	−0.0874	−0.0860
*Beauveria bassiana*			
PHI test	no recombination	n/p	recombination *
IAs NCC	1.0741 **	n/p	1.0455 ***
IAs CC	n/p	n/p	n/p

^1^ Abbreviations: dfb—warm-summer-humid continental climate zone; dfc—subarctic climate zone; NER—northern European Russia; NCC—non-clone-corrected data; CC—clone corrected data; n/p—not possible: calculation not done because of an insufficiently large dataset; ^2^ PHI (Φw, pairwise homoplasy index) test (Bruen et al., 2006) was calculated with SplitsTree 4 software (Huson, Bryant, 2006); ^3^ indexes of association (IAs) for non-clone-corrected and clone corrected data were calculated with LIAN 3.5 (Haubold, Hudson, 2000); significance values for the PHI test and IAs are as follows: * *p* < 0.05; ** *p* < 0.01; *** *p* < 0.001.

## Data Availability

The raw data analyzed during the study are available from the corresponding author on reasonable request. The obtained sequences were submitted in GenBank.

## Supplementary Materials

The following are available online at https://www.mdpi.com/article/10.3390/microorganisms9071409/s1, Table S1. *Beauveria* isolates information, Table S2. Accompanying species, Table S3. Haplotype polymorphism and recombination, Table S4. Spatial autocorrelation, Figure S1. Rarefaction curves for *Beauveria* spp., Figure S2. MLS phylogenetic tree of *Beauveria* spp., Figure S3. Percent of agricultural lands.
