# Supplementary material for: Distribution and Diversity of Beauveria in Boreal Forests of Northern European Russia
Source: Microorganisms. 2021 Jun 29;9(7):1409. doi: 10.3390/microorganisms9071409 (PMC8308049; doi:10.3390/microorganisms9071409)
Supplement: Supplementary file 1 [file microorganisms-09-01409-s001.zip › Figure_S1_Rarefaction_curves_for_Beauveria_spp..pdf]

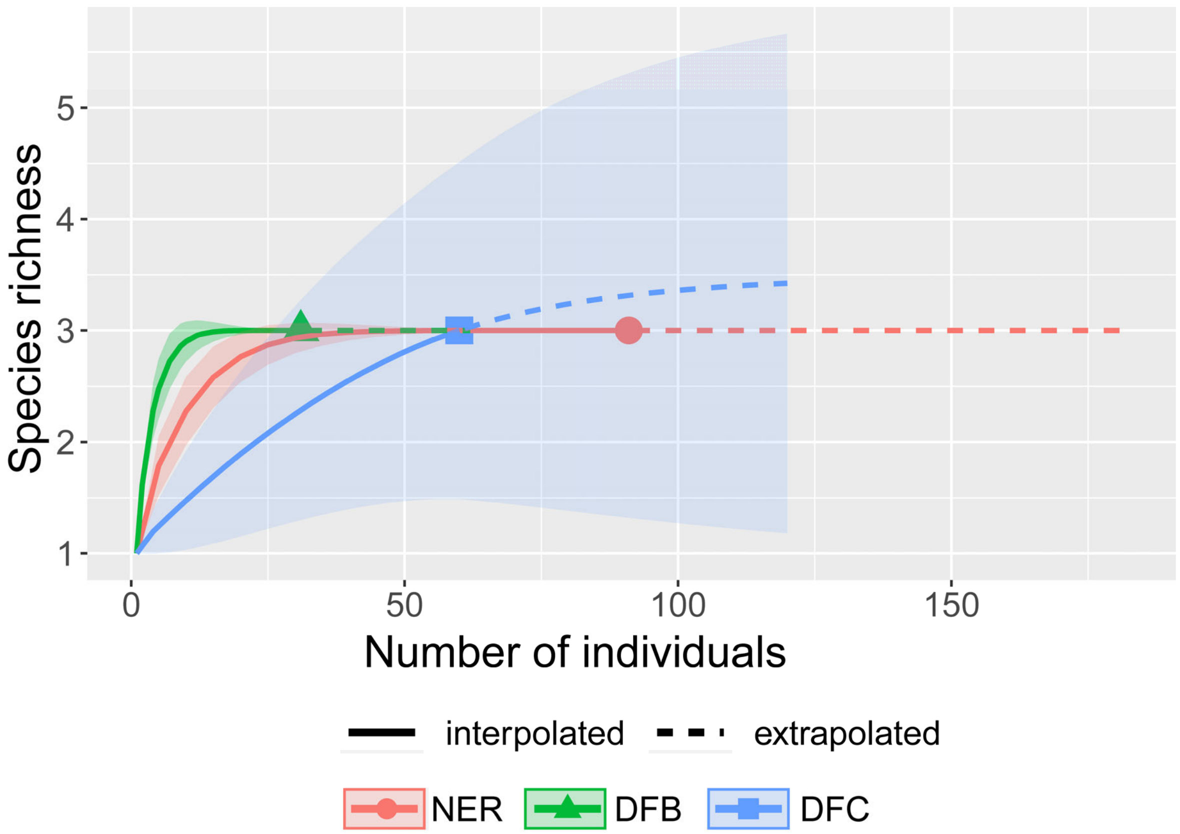

Figure S1. Rarefaction curves for total *Beauveria* species richness observed in Northern European Russia (NER) area and southern (dfb) and northern (dfc) subareas. Rarefaction curves for *B. bassiana* and *B. caledonica* were not obtained due to insufficient data. Transparent areas on plots corresponds to 95% confidence intervals.
