# Supplementary material for: Distribution and Diversity of Beauveria in Boreal Forests of Northern European Russia
Source: Microorganisms. 2021 Jun 29;9(7):1409. doi: 10.3390/microorganisms9071409 (PMC8308049; doi:10.3390/microorganisms9071409)

Figure S3. Percent of agricultural lands in each federal subject of Russian Federation. Data obtained from Analytical Center of the Russian Ministry of Agriculture (http://mcxac.ru/)

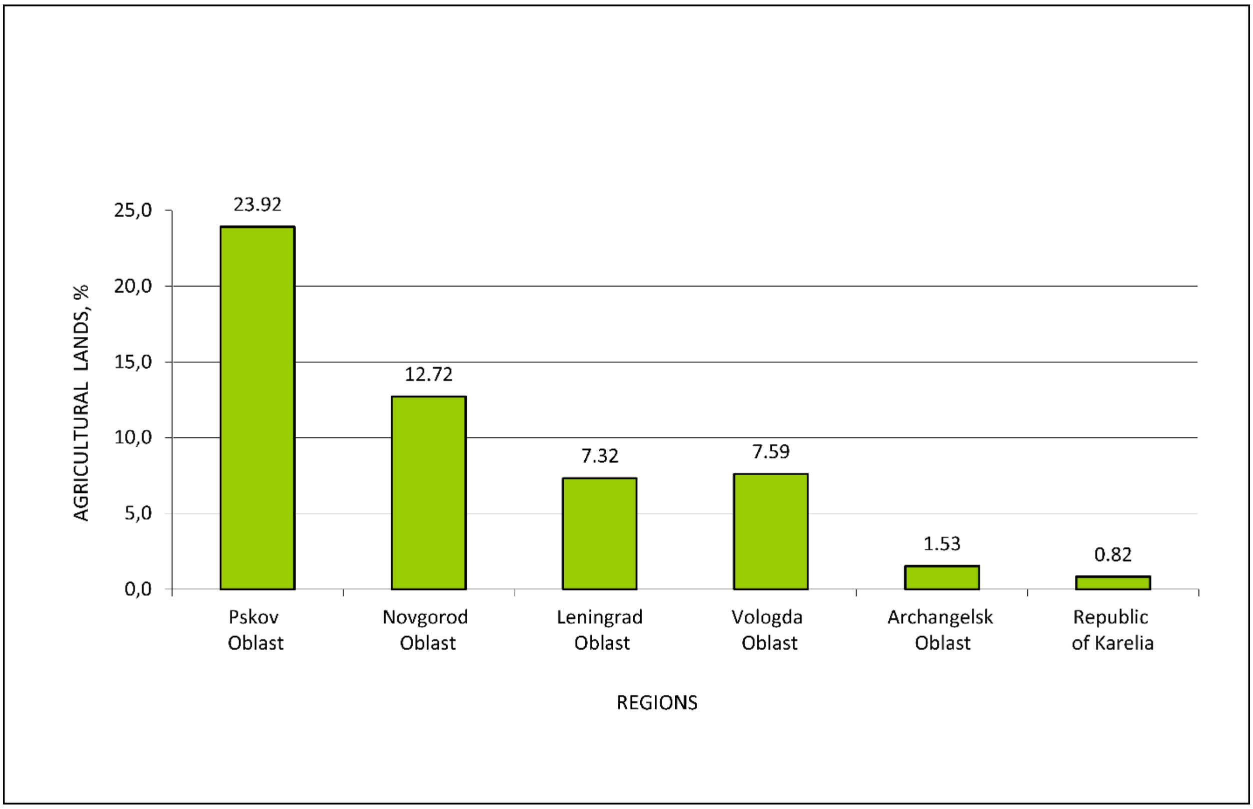

Supplement: Supplementary file 1 [file microorganisms-09-01409-s001.zip › Figure_S3_Percent_of_agricultural_lands.pdf]
