# Supplementary material for: Distribution and Diversity of Beauveria in Boreal Forests of Northern European Russia
Source: Microorganisms. 2021 Jun 29;9(7):1409. doi: 10.3390/microorganisms9071409 (PMC8308049; doi:10.3390/microorganisms9071409)
Supplement: Supplementary file 1 [file microorganisms-09-01409-s001.zip › Table_S3_Haplotype polymorphism and recombination .pdf]

Table S3. Haplotype polymorphism and recombination of *Beauveria* species

**Parameter**	**dfb**[1]	**dfc**	**NER**
B. pseudobassiana			
Number of isolates	17	57	74
Number of TEF alleles	3	3	4
TEF allelic diversity (SD)[2]	0.662(0.065)	0.628(0.034)	0.638(0.033)
Number of Bloc alleles	4	8	9
Bloc allelic diversity (SD)	0.566(0.123)	0.781(0.044)	0.738(0.045)
Number of MLS haplotypes	5	10	12
MLS allelic diversity (SD)	0.787(0.059)	0.872(0.02)	0.863(0.022)
Phylogenetic diversity	0.0155	0.0288	0.0326
PHI[3] test	1.0	0.010	0.007
IAs[4] NCC (p-value)	0.2928(0.00692)	0.2290(0.00007)	0.1970(0.00005)
IAs CC (p-value)	-0.0667(1)	-0.0874(1)	-0.0860(0.598)
B. bassiana			
Number of isolates	8	2	10
Number of TEF alleles	3	2	4
TEF allelic diversity (SD)	0.75(0.096)	1(0.5)	0,778 (0,091)
Number of Bloc alleles	3	2	4
Bloc allelic diversity (SD)	0.75(0.096)	1(0.5)	0,778(0.091)
Number of MLS haplotypes	3	2	4
MLS allelic diversity (SD)	0.75(0.096)	1(0.5)	0,778(0.091)
Phylogenetic diversity	0.0495	0.0064	0.0537
PHI test	1.0	n/p	0.025
IAs NCC (p-value)	1.0741(0.0035)	n/p	1.0455(0.00003)
IAs CC (p-value)	n/p	n/p	n/p
B. caledonica			
Number of isolates	6	1	7
Number of TEF alleles	1	1	1
TEF allelic diversity (SD)	0 (0)	n/p	0(0)
Number of Bloc alleles	1	1	2
Bloc allelic diversity (SD)	0 (0)	n/p	0.286(0.196)
Number of MLS haplotypes	1	1	2
MLS allelic diversity (SD)	0 (0)	n/p	0.286(0.196)
Phylogenetic diversity	0	0	0.0011
PHI test	n/p	n/p	n/p
IAs for NCC/CC (p-value)	n/p	n/p	n/p

[1] Abbreviations: dfb, warm-summer humid continental climate zone; dfc, subarctic climate zone; NER, northern European Russia; NCC, non-clone-corrected data; CC, clone corrected data; n/p, not possible: calculation not done because of insufficiently large dataset;

[2] Allelic (haplotype) diversity (Hd) and standard deviation (SD) were calculated for TEF, Bloc and multilocus data (MLS) with DNAsp (Librado, Rozas, 2009);

[3] PHI (Φw, pairwise homoplasy index) test (Bruen et al., 2006) was calculated with SplitsTree 4 software (Huson, Bryant, 2006);

[4] Indexes of association (IAs) for non-clone-corrected and clone corrected data were calculated with LIAN 3.5 (Haubold, Hudson, 2000);
