# Supplementary figures and images for: Distribution and Diversity of Beauveria in Boreal Forests of Northern European Russia
Source: Microorganisms. 2021 Jun 29;9(7):1409. doi: 10.3390/microorganisms9071409 (PMC8308049; doi:10.3390/microorganisms9071409)

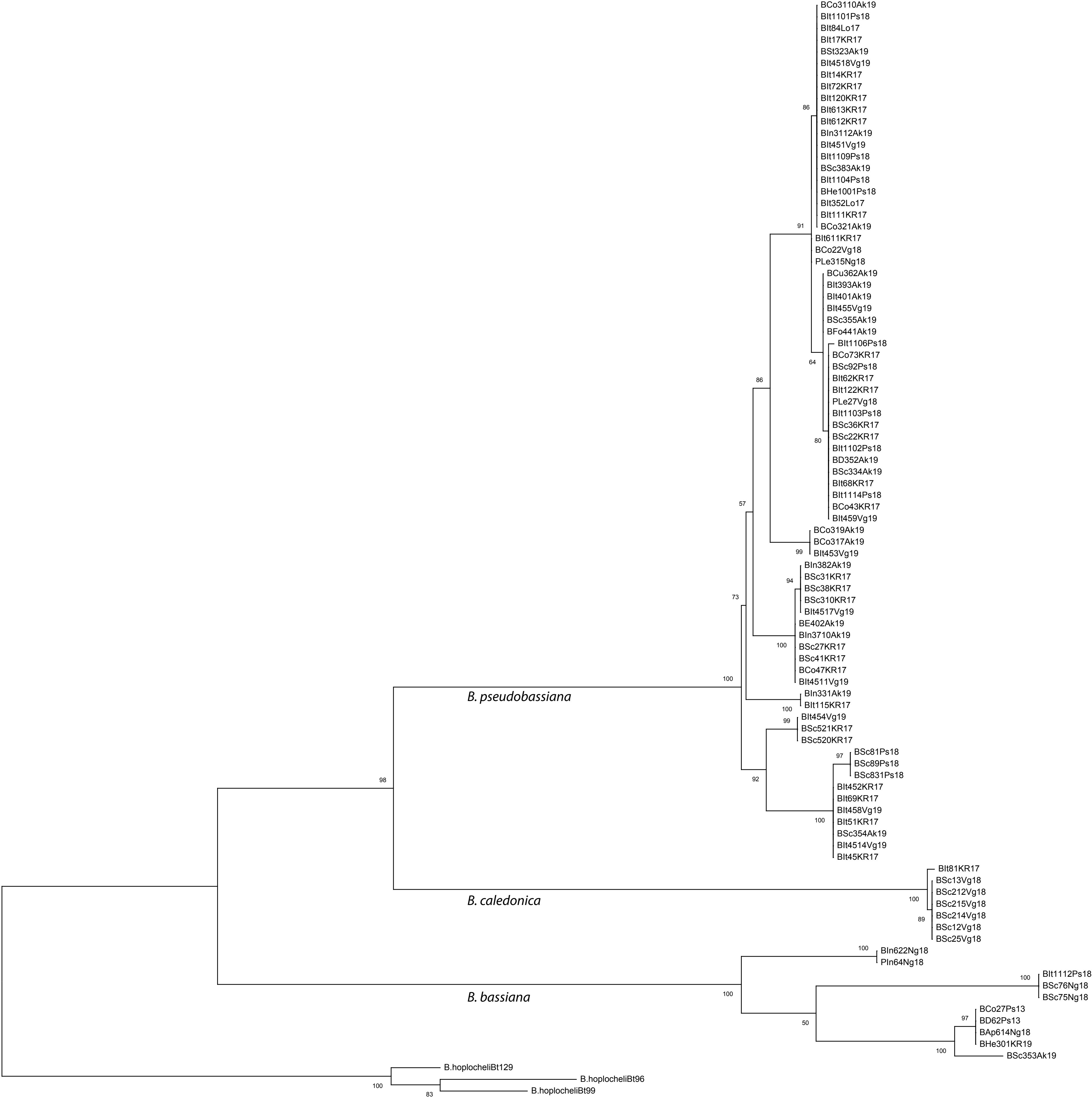

0.020

Supplement: Supplementary file 1 [file microorganisms-09-01409-s001.zip › Figure_S2_MLS phylogenetic tree of Beauveria spp..pdf]
